# Clinical determinants of hospital treated deliberate self‐harm repetition: A time to recurrent event analysis

**DOI:** 10.1111/acps.13503

**Published:** 2022-10-09

**Authors:** Anne Seljenes Bøe, Lars Mehlum, Ingrid Melle, Ping Qin

**Affiliations:** ^1^ The National Centre for Suicide Research and Prevention Institute of Clinical Medicine, University of Oslo Oslo Norway; ^2^ Division of Mental Health and Addiction Oslo University Hospital and Institute of Clinical Medicine Oslo Norway

**Keywords:** deliberate self‐harm repetition, patient registry, population study, psychiatric disorders

## Abstract

**Introduction:**

Psychiatric disorders are strongly associated with hospital treated deliberate self‐harm (DSH). However, the effect of specific disorders on risk for DSH repetition in sex‐age‐subgroups is understudied. The present study aims to assess the influence of various specific psychiatric disorders on risk for subsequent DSH repetition by way of time to recurrent event analysis on a national cohort of DSH patients.

**Material and Methods:**

Individuals aged 18 and older presenting to somatic hospital for DSH during the period 2008–2018 was identified through national registers. A parametric shared frailty survival analysis was used to investigate the impact of various psychiatric disorders on risk of DSH repetition.

**Results:**

The cohort consisted of 39,508 individuals of which 8634 (21.8%) presented with 24,028 repeated episodes of hospital treated DSH. Borderline personality disorder increased the risk of DSH repetition in females (adjusted HR 1.49, CI 1.41–1.57), while alcohol use disorder (HR 1.12, CI 1.04–1.19) and substance use disorders (adjusted HR 1.22, CI 1.14–1.32) increased the risk of repetition in males. The strongest impact of psychiatric disorder on risk of repetition was found among the elderly. Previous history of DSH was associated with the highest increased risk of repetition.

**Conclusion:**

Prior history of DSH was strongly associated with DSH repetition, but the influence of psychiatric disorder varied significantly by specific diagnoses and by sex and age of the patients. Efforts to prevent DSH repetition should be age and gender specific and designed to meet the needs of people with different specific psychiatric disorders.

**Significant outcomes**

- The effect of psychiatric disorders on risk for hospital treated DSH repetition varies greatly by age and gender of the patient.
- Borderline personality disorder increased the risk of DSH repetition in younger females, while alcohol use disorder increased the risk of DSH repetition in middle‐aged and elderly males.
- The association between DSH repetition associated with psychiatric disorders was strongest among the elderly.

**Limitations**

- The cohort studied was limited to individuals residing in Norway during the complete study period and variables available through the national registers used.
- Data on whether or not a psychiatric assessment was performed during admission to general hospital was not available through the register data.

## 1 INTRODUCTION

Repetition of deliberate self‐harm (DSH) is common. A recent systematic review found a pooled repetition rate of 17% within 1 year and 24% within 3 years of follow‐up.^1^ Important clinical risk factors for DSH repetition among hospital treated DSH patients include personality disorders, substance use disorders, depression and schizophrenia.^2^, ^3^ Other risk factors for DSH repetition include previous history of DSH and psychiatric treatment, living alone, hopelessness and self‐injury as method of self‐harm.^3^, ^4^ Although large‐scale register studies on DSH repetition treated in general hospital have been performed,^5^ few have focused on specific psychiatric disorders, resulting in a lack of knowledge of specific clinical risk factors for repetition in this patient population.

Another less emphasized area of research is age‐specific risk factors for DSH repetition, especially in middle age and older age. It is essential to take into account the impact of age on DSH repetition, as DSH and DSH repetition are associated with an increased risk of suicide not only among the young but among older age groups as well.^6^ Moreover, detailed knowledge of age and gender specific clinical risk factors in this heterogeneous patient population is needed to facilitate targeted prevention of DSH repetition.

History of DSH is reportedly the strongest risk factor for future DSH repetition in several previous studies.^5^, ^7^ Among individuals with no previous history of DSH, on the other hand, identification of risk factors other than personality disorders are limited.^7^

The aims of this study were, therefore, twofold. Firstly, we wanted to profile repeaters of hospital‐treated DSH and non‐repeaters to examine potential sociodemographic and clinical differences between the groups. Secondly, we aimed to assess the influence of specific psychiatric disorders diagnosed during hospital treatment for DSH on the risk of subsequent DSH repetition by way of time to recurrent event analysis using register data from a large national cohort. We hypothesize that the effect of psychiatric disorders on DSH repetition differs by specific diagnoses, and by sex and age of the patients.

## 2 MATERIALS AND METHODS

### 2.1 Data sources

The study was based on data from three Norwegian longitudinal registers that were interlinked on an individual level via the encrypted personal identifier of all residents in Norway. The Central Population Register, administered by the Norwegian Tax Administration, contains demographic data such as citizenship, gender and date of birth. The Statistics Norway's Events Databases (FD‐trygd), administrated by Statistics Norway (SSB), contains socioeconomic data such as marital status, annual income and educational attainment. The Norwegian Patient Register (NPR), administered by the Directorate of Health and computerized since 2008, provides a wide range of clinical information from hospital treatment as well as specialized treatment outside of the hospital. All medical diagnoses are provided with codes according to ICD‐10.

### 2.2 Study population

From the NPR we identified a cohort of all individuals aged 18 or older with one or more DSH episodes treated in Norwegian general hospital services between January 1st, 2008 and December 31st, 2018. DSH was defined as “intentional self‐poisoning or self‐injury, irrespective of motivation”.^8^ Because of the underreporting of DSH in the patient registry, we adopted a broader approach to include episodes of probable DSH, by applying a sophisticated process of data selection and a data‐driven algorithm as described in full detail by Qin and Mehlum.^9^ Briefly, we first identified all episodes of injury and poisoning that received urgent treatment and that were at least one day apart from any previous episode. All indirect contacts, planned treatments, fatal injuries and poisonings or injuries that were clearly accidental or inflicted by others, or that were secondary outcomes of other medical conditions were excluded from consideration as being ineligible by the definition of DSH. Based on the examination of data on incidents which were given a diagnosis of DSH (ICD‐code: X6n, Y87), we then undertook three steps in a hierarchical procedure to ascertain probable DSH episodes. The first step was to include treatment contacts because of injuries with a diagnosis of DSH (ICD‐code: X6n, Y87). The second step was to include treatment contacts that had a diagnosis of either poisoning with the ICD‐10 codes T4n, T50‐T55, T57‐T60, T62, T65 or T96, or injuries with the ICD‐10 codes S10‐S11, S15, S19, S21, S25‐S27, S31, S35‐S39, S41, S45, S50‐S51, S54‐S56, S59, S61, S64‐S65, S69, S71, S88, T01, T09, T11, T18‐T19, T27‐T28, T31, T68, T69, T71 or T95, if they had a comorbid psychiatric diagnosis (ICD‐10 codes F0‐F9). The final step was to include treatment contacts with poisoning (ICD‐10 codes T4n, T50 and T96) that were not covered by the previous 2 steps. After these procedures of selection, 39,518 individuals with 63,552 episodes of hospital treated DSH were included, covering both injury and poisoning among Norwegian adults. Retrieval of data from source registers and construction of the data set for analysis were conducted using SAS/STAT software, version 9.4 of the SAS System for Windows.

### 2.3 Variables

Clinical data, all extracted from the NPR, included hospital treatment of DSH, psychiatric disorder diagnosed during hospital treatment for DSH, DSH method and number of DSH episodes (with each episode's sequence number) within the study period. Psychiatric disorder diagnosed during hospital treatment for DSH was categorized according to ICD‐10 as follows: “Alcohol use disorders (F10)”, “Substance use disorders (F11‐19)”, “Schizophrenia spectrum disorders (F20‐29)”,” Bipolar spectrum disorders (F30‐31)”, “Depressive disorders (F32‐39)”, “Anxiety disorders (F40‐42)”, “PTSD and other stress related disorders (F43)”, “Eating disorders (F50)”, Borderline personality disorder (F60.3)”, “Other personality disorders (F60‐69, excluding F60.3)” and “Any other psychiatric disorder (F44‐48, F51‐59, F70‐99)”. These diagnoses were retrieved based on data of primary and secondary diagnoses and therefore not mutually exclusive, meaning that a patient could have more than one psychiatric disorder at any given DSH episode. Number of psychiatric diagnoses was further summarized as “No psychiatric disorders”, “1 psychiatric disorder”, “2 psychiatric disorders” and “3 or more psychiatric disorders” for each DSH episode. Method of DSH was categorized into “Injury”, “Poisoning”, or a combination of the two labeled as “Mixed method”. Total number of DSH episodes were coded as follows: “1”, “2”, “3–5”, “6–10” and “≥11”. Grouped in the same way, number of previous DSH episodes was used in the multivariate analysis to control for any previous DSH episodes by the individual during the observation period. Length of hospital stay was coded in days as follows: “0”, “1”, “2” and “≥3”.

Sociodemographic information was extracted from the Central Population Register and the FD‐Trygd Databases. Variables of interest were gender, age, marital status, educational attainment, level of income and ethnicity. Age was coded as “18–24”, “25–39”, “40–64” and “≥65”. Marital status was coded as “married”, “unmarried”, “separated”, “divorced” and “widowed”. Cohabiting couples were coded as unmarried. Educational attainment was coded as “compulsory education”, “high school”, “vocational education” and “higher education (i.e., bachelor or higher levels of education)”. Level of income was coded as “0–2G”, “2–3G”, “3–4G”, “4–6G” and “≥6G”. 1G is a constant, denoting the base amount used in the Norwegian public pension system to calculate pension and other public benefits, and is adjusted yearly according to the growth in annual income. In 2008, the base amount G was 70,256 NOK, in 2018 the base amount was 96,883 NOK. Median income per capita in Norway fluctuates between 3G and 4G. Data regarding annual income was collected the year before DSH treatment. Ethnicity was coded as “Ethnic Norwegian” covering all Norwegian born individuals with Norwegian born parents, “1^st^ generation immigrant” containing foreign born individuals with foreign born parents. Foreign born individuals with one Norwegian born parent and Norwegian born individuals with one foreign born parent together with 2nd generation immigrants were labeled as “Mixed background”.

### 2.4 Statistical analysis

Chi‐square tests were used to examine the differences in variable distribution between non‐repeaters and repeaters at index episode of DSH. A shared parametric frailty survival analysis was conducted to assess the influence of specific psychiatric disorders diagnosed in prior DSH episode on risk for subsequent DSH repetition in the context of other clinical covariates. A shared frailty survival analysis introduces a random latent effect into the survival model to account for the intra‐subject correlation that arises with recurrent event data.^10^ All DSH episodes after the first episode by the same individual were modeled as recurrent events in the following way: calculation of time was based on the date of DSH treatment and counted in days from the first episode to the second episode, from the second to the third and so forth until the last DSH episode. This data structure considers each individual at risk of DSH repetition after their first episode until their first repetition or censoring, and after that from their first repetition until a second repetition or censoring and so forth. Consequently, this data structure requires that the analysis is adjusted for number of previous DSH episodes by the individual during the observation period. Individuals were censored on date of death, emigration or study stop. The data structure required for time to recurrent event models and syntax for the analysis used in the present article are both described in detail by Amorim and Cai^11^ (in web appendix 1 and 2). All analyses were performed using Stata MP 16.1 for Windows.

## 3 RESULTS

### 3.1 Distribution of sociodemographic variables

The present cohort consisted of 39,508 patients with a total of 63,536 hospital treated DSH episodes. In all, 8634 patients (21.8%) were recorded with 24,028 repeated episodes. 6975 patients were censored because of death and 718 patients were censored because of emigration during the study period. The total number of DSH episodes per patient ranged from 1 to 218 among females and 1 to 82 among males. The 4819 episodes numbered 11th and above were by only 356 patients, comprising 55 patients aged 18–24, 191 patients aged 25–39, 104 patients aged 40–64 and 6 patients over 64 years old and 88.5% of the episodes were by females.

The distribution of all sociodemographic variables differed significantly between non‐repeaters and repeaters at the index episode of DSH (Table 1). The majority of the DSH patients in the present cohort was unmarried, had low education and income and was ethnic Norwegians. However, compared with the non‐repeaters at the index episode of DSH, repeaters were less often married or widowed and were of younger age.

**TABLE 1:** Distribution of demographic variables by non‐repeaters. Repeaters and by number of repetitions^a^

	Non‐repeaters		Repeaters									
	Index episode		Index episode		2nd		3rd–5th		6th–10th		≥11	
	(*n* = 30,874)		(*n* = 8634)		(*n* = 8634)		(*n* = 7270)		(*n* = 3305)		(*n* = 4819)	
Gender												
Female	16,451	53.3	4993	57.8	4994	57.8	4723	65.0	2569	77.7	4263	88.5
Male	14,423	46.7	3640	42.2	3640	42.2	2547	35.0	736	22.2	556	11.5
Age group												
18–24	6568	21.3	2332	27.0	1961	22.7	1741	23.9	930	28.1	1053	21.8
25–39	7555	24.5	2582	29.9	2681	31.0	2544	34.9	1262	38.2	2732	56.7
40–64	10,205	33.0	3134	36.3	3302	38.2	2709	37.3	1040	31.5	983	20.4
≥65	6546	21.2	586	6.8	690	8.0	276	3.8	73	2.2	51	1.1
Marital status												
Married	6414	20.8	1212	14.0	1064	12.3	608	8.4	166	5.0	132	2.7
Unmarried	15,681	50.8	5353	62.0	5300	61.4	4881	67.1	2464	74.5	4209	87.3
Separated	1200	3.9	406	4.7	430	5.0	377	5.2	171	5.2	132	2.7
Divorced	4761	15.3	1402	16.2	1547	17.9	1275	17.5	465	14.0	336	7.0
Widowed	2702	8.8	249	2.9	289	3.3	126	1.7	38	1.1	10	0.2
Education												
Compulsory education	15,247	49.4	4941	57.2	4851	56.2	4202	57.8	2000	60.5	2939	61.0
High school	3775	12.2	760	8.8	759	8.8	464	6.4	160	4.8	140	2.9
Vocational education	6834	22.1	1789	20.7	1845	21.4	1633	22.5	755	22.8	1225	25.4
Higher education	3838	12.4	862	10.0	905	10.5	761	10.5	310	9.4	306	6.3
Income												
0–2G	7294	23.6	2364	27.4	1965	22.8	1539	21.2	570	17.2	437	9.1
2–3G	8723	28.3	2646	30.6	3014	34.9	2870	39.5	1523	46.1	3025	62.7
3–4G	6381	20.7	1764	20.4	1926	22.3	1690	23.3	805	24.4	1116	23.1
4–6G	5888	19.1	1401	16.2	1328	15.4	947	13.0	348	10.5	218	4.5
≥6G	2526	8.2	450	5.2	395	4.6	215	3.0	54	1.6	15	0.3
Immigrant status												
Ethnic Norwegian	25,291	81.9	7431	86.1	7431	86.1	6470	89.0	2998	90.7	4421	91.7
1st generation immigrant	3487	11.3	571	6.6	571	6.6	297	4.1	72	2.2	25	0.5
2nd gen./mixed background	2096	6.7	632	7.3	632	7.3	503	6.9	235	7.1	373	7.7

^a^ Significant *X* ^*2*^ tests (*p* < 0.01) for all variables between non‐repeaters and repeaters at index episode of DSH.

### 3.2 Distribution of clinical variables

Psychiatric morbidity was higher among the repeaters (58.1%) than the non‐repeaters (47.6%). Depressive disorders, alcohol use disorder and substance use disorders were the most common disorders among repeaters and non‐repeaters. Borderline personality disorder displayed the largest increase in representation among the repeated episodes, from 2.9% at the index episode to 42.2% in the high‐volume repeat episodes (Table 2). Depressive disorders displayed the largest decrease in prevalence across the repeated episodes, from 18.9% in the index to 4.2% in the high‐volume repeat episode group. Overall, poisoning as the most frequent method of self‐harm, but self‐injury as method of DSH increased from 8.8% of the index episodes among the repeaters to 28.2% of the high‐volume repetitions.

**TABLE 2:** Distribution of clinical variables by non‐repeaters, repeaters and by number of repetitions

	Non‐repeaters^a^		Repeaters									
	Index episode		Index episode		2nd		3rd–5th		6th–10th		≥11	
	(*n* = 30,873)		(*n* = 8634)		(*n* = 8634)		(*n* = 7270)		(*n* = 3305)		(*n* = 4819)	
Psychiatric disorder^b^												
Alcohol use disorders	5018	16.2	1511	17.5	1603	18.6	1308	18.0	515	15.6	456	9.5
Substance use disorders	2740	8.9	1094	12.7	1237	14.3	1065	14.6	376	11.4	315	6.5
Schizophrenia spectrum disorders	858	2.8	293	3.4	320	3.7	268	3.7	141	4.4	161	3.3
Bipolar spectrum disorders	931	3.0	353	4.1	406	4.7	301	4.1	141	4.3	242	5.0
Depressive disorders	4844	15.7	1635	18.9	1382	16.0	983	13.5	327	9.9	201	4.2
Anxiety disorders	906	2.9	326	3.8	344	4.0	282	3.9	93	2.8	88	1.8
PTSD and other stress related disorders	628	2.0	184	2.1	247	2.9	290	4.0	186	5.6	223	4.6
Eating disorders	122	0.4	94	1.1	85	1.0	115	1.6	84	2.5	69	1.4
Borderline personality disorder	242	0.8	248	2.9	343	4.0	734	10.1	704	21.3	2036	42.2
Any other personality disorder	243	0.8	191	2.2	239	2.7	368	5.1	252	7.6	517	10.7
Any other psychiatric disorder	766	2.5	270	3.1	310	3.6	287	3.9	211	6.4	260	5.4
Number of psychiatric disorders												
No psychiatric disorders	16,191	52.4	3622	41.9	3456	40.0	2610	35.9	1085	32.8	1321	27.4
1 disorder	12,336	40.0	3980	46.1	4035	46.7	3527	47.0	1555	47.0	2571	53.3
2 disorders	2109	6.8	887	10.3	976	11.3	956	13.1	528	16.0	795	16.5
3 or more disorder	238	0.7	145	1.7	167	1.9	177	2.4	127	4.1	132	2.7
DSH method												
Poisoning	25,605	82.9	7674	88.9	7534	87.3	6392	87.9	2841	85.9	3301	68.5
Injury	4437	14.4	758	8.8	862	10.0	699	9.6	360	10.9	1359	28.2
Mixed method	832	2.7	202	2.3	238	2.8	179	2.5	104	3.1	159	3.3
Length of hospital stay (days)												
0	8959	29.0	2629	30.4	2595	30.0	2234	30.7	1061	32.1	1620	33.6
1	12,222	39.6	3833	44.4	3774	43.7	3499	48.1	1606	48.6	2217	46.0
2	3541	11.5	1049	12.2	1044	12.1	815	11.2	386	11.7	524	10.9
≥3	6152	19.9	1123	13.0	1221	14.1	722	9.9	252	7.6	458	9.5

^a^ Significant *X* ^2^ tests (*p* < 0.01) for all variables except “PTSD and other stress related disorders” and “Eating disorders” between non‐repeaters and repeaters at index episode of DSH.

^b^ ^“^Alcohol use disorders”: F10, “Substance use disorders”: F11‐19, “Schizophrenia spectrum disorders”: F20‐29, “Bipolar spectrum disorders”: F30‐31, “Depressive disorders”: F32‐39, “Anxiety disorders”: F40‐42, “PTSD and other stress related disorders”: F43, “Eating disorders”: F50, “Borderline personality disorder”: F60.3, “Any other personality disorder”: F60‐69 excluding F60.3 and “Any other psychiatric disorder”: F44‐48, F51‐59, F70‐99.

### 3.3 Association between clinical variables and DSH repetition overall and by gender

Of all the psychiatric disorders borderline personality disorder (HR 1.49, CI 1.41–1.57) and other personality disorders, mostly unspecified personality disorders (F60.9) (HR 1.22, CI 1.14–1.31), displayed the strongest association with DSH repetition in the adjusted analysis together with bipolar spectrum disorder (HR 1.14, CI 1.05–1.24) (Table 3). Having 11 or more previous DSH episodes displayed the strongest association (HR 6.83, CI 6.23–7.49) with DSH repetition. The age group “25–39” displayed the highest risk of repetition (HR 1.99, CI 1.80–2.20). Females (HR 1.36, CI 1.30–1.43) displayed an increased overall risk of repetition compared with males.

**TABLE 3:** Frailty survival analysis of the association between clinical factors and DSH repetition overall and by gender

	Crude^b^	Adjusted^c^	Female^c^	Male^c^
	HR (CI)	HR (CI)	Adjusted HR (CI)	Adjusted HR (CI)
Psychiatric disorder^a^				
Alcohol use disorder	1.21 (1.05–1.40)	0.99 (0.94–1.04)	0.90 (0.84–0.96)*	1.12 (1.04–1.19)*
Substance use disorders	1.13 (1.06–1.20)*	1.03 (0.98–1.09)	0.92 (0.85–0.99)	1.22 (1.14–1.32)*
Schizophrenia spectrum disorders	1.11 (1.01–1.23)	1.03 (0.94–1.13)	1.11 (0.99–1.23)	0.87 (0.74–1.02)
Bipolar spectrum disorders	1.26 (1.14–1.39)*	1.14 (1.05–1.24)*	1.14 (1.04–1.26)*	1.15 (0.98–1.35)
Depressive disorders	1.03 (0.97–1.09)	1.05 (1.00–1.10)	1.04 (0.98–1.10)	1.09 (1.00–1.19)
Anxiety disorders	1.08 (0.98–1.20)	1.05 (0.96–1.15)	1.02 (0.91–1.14)	1.10 (0.97–1.29)
PTSD and other stress related disorders	1.26 (1,15–1.38)*	1.01 (0.93–1.10)	1.02 (0.93–1.12)	0.99 (0.81–1.22)
Eating disorders	1.66 (1.58–1.75)*	1.05 (0.92–1.21)	1.00 (0.87–1.16)	2.59 (1.04–6.43)
Borderline personality disorder	1.95 (1.84–2.06)*	1.49 (1.41–1.57)*	1.49 (1.41–1.57)*	1.20 (0.99–1.47)
Other personality disorders	1.26 (1.16–1.35)*	1.22 (1.14–1.31)*	1.23 (1.14–1.33)*	1.13 (0.95–1.34)
Any other psychiatric disorder	1.37 (1.26–1.50)*	1.11 (1.02–1.20)*	1.11 (1.02–1.21)*	1.08 (0.92–1.27)
Previous psychiatric disorder				
No	‐	‐	‐	‐
Yes	1.21 (1.17–1.26)*	1.07 (1.03–1.20)*	1.07 (1.02–1.12)*	1.07 (0.98–1.16)
Length of hospital stay (days)				
0	1.43 (1.35–1.53)*	1.21 (1.14–1.28)*	1.33 (1.24–1.43)*	1.00 (0.91–1.10)
1	1.52 (1.44–1.61)*	1.29 (1.22–1.36)*	1.40 (1.31–1.50)*	1.09 (1.01–1.20)
2	1.46 (1.36–1.57)*	1.28 (1.20–1.36)*	1.35 (1.25–1.47)*	1.16 (1.04–1.30)*
≥3	‐	‐	‐	‐
Previous DSH episodes				
0	‐	‐	‐	‐
1	1.77 (1.67–1.89)*	1.72 (1.58–1.95)*	1.80 (1.66–1.95)*	1.70 (1.51–1.91)*
2	2.39 (2.21–2.58)*	2.25 (2.08–2.44)*	2.40 (2.18–2.64)*	2.14 (1.84–2.48)*
3–5	3.54 (3.28–3.83)*	3.20 (2.96–3.47)*	3.44 (3.14–3.78)*	2.95 (2.52–3.45)*
6–10	5.03 (4.61–5.48)*	4.30 (3.93–4.70)*	4.44 (4.01–4.92)*	4.29 (3.54–5.18)*
11+	8.47 (7.76–9.24)*	6.83 (6.23–7.49)*	7.07 (6.37–7.85)*	6.80 (5.50–8.42)*
Method of DSH				
Poisoning	‐	‐	‐	‐
Injury	0.95 (0.90–1.00)	0.91 (0.86–0.95)*	1.00 (0.94–1.07)	0.70 (0.64–0.76)*
Mixed method	0.96 (0.87–1.06)	0.96 (0.87–1.05)	1.03 (0.92–1.15)	0.81 (0.69–0.96)
Age group				
18–24	3.31 (3.00–3.65)*	1.75 (1.58–1.95)*	1.89 (1.65–2.17)*	1.45 (1.22–1.71)*
25–39	3.76 (3.42–4.14)*	1.99 (1.80–2.20)*	2.15 (1.89–2.45)*	1.78 (1.53–2.08)*
40–64	2.82 (2.56–3.10)*	1.86 (1.70–2.04)*	1.84 (1.63–2.08)*	1.88 (1.64–2.16)*
≥65	‐	‐	‐	‐
Gender				
Female	1.73 (1.64–1.83)*	1.36 (1.30–1.43)*	‐	‐
Male		‐	‐	‐

^a^ Alcohol use disorders”: F10, “Substance use disorders”: F11‐19, “Schizophrenia spectrum disorders”: F20‐29, “Bipolar spectrum disorders”: F30‐31, “Depressive disorders”: F32‐39, “Anxiety disorders”: F40‐42, “PTSD and other stress related disorders”: F43, “Eating disorders”: F50, “Borderline personality disorder”: F60.3, “Any other personality disorder”: F60‐69 excluding F60.3 and “Any other psychiatric disorder”: F44‐48, F51‐59, F70‐99.

^b^ Psychiatric disorders adjusted for any other psychiatric disorder.

^c^ Controlled for marital status, education, income and ethnicity. * Significance at 0.01.

Among females, borderline personality disorder (HR 1.49, CI 1.41–1.57), any other personality disorder (HR 1.23, CI 1.14–1.33) and bipolar spectrum disorders (HR 1.14, CI 1.04–1.26) increased the risk of DSH repetition whereas alcohol use disorder was negatively associated with DSH repetition (HR 0.90, CI 0.84–0.96). In males, substance use disorders (HR 1.22, CI 1.14–1.32) and alcohol use disorder (HR 1.12, CI 1.04–1.19) were significantly associated with DSH repetition. Moreover, females and males with 11 or more previous episodes of DSH displayed a close to sevenfold risk of further DSH repetition (females HR 7.07, CI 6.37–7.85, males HR 6.80, CI 5.50–8.42). Females aged 25–39 (HR 2.15, CI 1.89–2.45) and males aged 40–64 (HR 1.88, CI 1.64–2.16) displayed the strongest association with DSH repetition.

### 3.4 Association between clinical variables and DSH repetition by age group

Borderline personality disorder significantly increased the risk of DSH repetition in females in the age groups “18–24” (HR 1.43 CI 1.32–1.55) and “25–39” (HR 1.58, CI 1.44–1.74) (Table 4). Any other personality disorder increased the risk of DSH repetition in females in the age groups “18–24” (HR 1.34, CI 1.18–1.51) and “40–64” (HR 1.53, CI 1.24–1.90). Substance use disorders were associated with an increased risk of repetition in males aged “25–39” (HR 1.35, CI 1.21–1.51) and over 65 (HR 2.28, CI 1.22–4.26). Alcohol use disorder was associated with an increased risk of repetition among middle‐aged and elderly males (HR 1.20, CI 1.09–1.33 males “40–64”, HR 1.68, CI 1.21–2.34 males “≥65”). Bipolar spectrum disorders (HR: 2.58, CI 1.75–3.81) were associated with a significantly increased risk of DSH repetition only in females over the age of 65. Depressive disorders increased the risk of repetition among the elderly (HR females 1.83, CI 1.45–2.32, HR males 1.94, CI 1.36–2.78). However, caution is needed when interpreting the significant findings in the oldest age group and regarding eating disorders in males, as the number of repeated episodes of DSH with the respective psychiatric diagnoses was very small in this age group.

**TABLE 4:** Frailty survival analysis of the association between psychiatric disorders and DSH repetition by age group and gender, HR(CI)

	18–24^b^	25–39^b^	40–64^b^	≥65^b^
Total, *n* (repeater/non‐repeater)	8900 (2332/6568)	10,137 (2582/10205)	13,339 (3134/10205)	7132 (586/5646)
Psychiatric disorder^a^				
Alcohol use disorders	0.90 (0.81–1.00)	0.87 (0.80–0.95)*	1.10 (1.03–1.18)*	1.59 (1.25–2.02)*
Substance use disorders	0.86 (0.78–0.95)*	1.15 (1.06–1.25)*	1.05 (0.95–1.15)	1.55 (1.07–2.25)*
Schizophrenia spectrum disorders	1.04 (0.87–1.24)	0.95 (0.81–1.10)	1.11 (0.96–1.29)	1.27 (0.71–2.26)
Bipolar spectrum disorders	1.13 (0.95–1.34)	1.18 (1.01–1.36)	1.05 (0.92–1.20)	2.33 (1.66–3.27)*
Depressive disorders	1.00 (0.91–1.10)	1.00 (0.91–1.09)	1.05 (0.97–1.14)	1.86 (1.53–2.27)*
Anxiety disorders	0.95 (0.77–1.17)	0.94 (0.79–1.12)	1.12 (0.99–1.28)	1.61 (1.15–2.25)*
PTSD and other stress related disorders	1.11 (0.97–1.26)	0.92 (0.80–1.06)	1.00 (0.82–1.21)	1.24 (0.54–2.83)
Eating disorders	0.96 (0.81–1.14)	1.06 (0.81–1.39)	1.32 (0.77–2.26)	‐
Borderline personality disorder	1.41 (1.31–1.52)*	1.60 (1.47–1.75)*	1.25 (1.08–1.45)*	1.32 (0.35–5.01)
Other personality disorders	1.29 (1.15–1.45)*	1.10 (0.98–1.23)	1.40 (1.19–1.65)*	2.23 (0.87–5.72)
Any other psychiatric disorder	1.11 (0.99–1.24)	1.04 (0.90–1.21)	1.09 (0.92–1.34)	1.49 (0.82–2.72)
Females, *n* (repeater/non‐repeater)	5586 (1631/3955)	4945 (1333/3612)	6820 (1661/5159)	4094 (369/3725)
Psychiatric disorder^a^				
Alcohol use disorders	0.83 (0.72–0.95)*	0.85 (0.76–0.96)*	1.01 (0.91–1.12)	1.28 (0.86–1.89)
Substance use disorders	0.82 (0.73–0.92)*	0.97 (0.86–1.09)	0.97 (0.84–1.13)	1.30 (0.82–2.07)
Schizophrenia spectrum disorders	1.04 (0.85–1.26)	1.04 (0.86–1.25)	1.30 (1.08–1.58)*	0.93 (0.41–2.09)
Bipolar spectrum disorders	1.14 (0.95–1.38)	1.20 (1.02–1.43)	1.02 (0.86–1.20)	2.58 (1.75–3.81)*
Depressive disorders	0.97 (0.87–1.08)	0.98 (0.88–1.10)	1.08 (0.98–1.20)	1.83 (1.45–2.32)*
Anxiety disorders	0.90 (0.71–1.15)	0.72 (0.57–0.93)*	1.24 (1.05–1.47)*	1.68 (1.14–2.47)*
PTSD and other stress related disorders	1.11 (0.97–1.27)	0.93 (0.78–1.10)	0.95 (0.74–1.21)	0.95 (0.28–3.23)
Eating disorders	0.96 (0.81–1.14)	0.98 (0.74–1.30)	1.16 (0.66–2.03)	‐
Borderline personality disorder	1.43 (1.32–1.55)*	1.58 (1.44–1.74)*	1.24 (1.05–1.46)	1.55 (0.39–5.95)
Other personality disorders	1.34 (1.18–1.51)*	1.07 (0.95–1.20)	1.53 (1.24–1.90)*	1.79 (0.62–5.18)
Any other psychiatric disorder	1.05 (0.93–1.19)	1.12 (0.95–1.33)	1.15 (0.90–1.46)	1.72 (0.87–3.38)
Males, *n* (repeater/non‐repeater)	3314 (701/2613)	5192 (1249/3943)	6519 (1473/5046)	3083 (217/2821)
Psychiatric disorder^a^				
Alcohol use disorders	1.12 (0.94–1.33)	0.93 (0.82–1.06)	1.20 (1.09–1.33)*	1.68 (1.21–2.34)*
Substance use disorders	0.99 (0.83–1.18)	1.35 (1.21–1.51)*	1.12 (0.99–1.27)	2.28 (1.22–4.26)*
Schizophrenia spectrum disorders	1.02 (0.65–1.59)	0.75 (0.57–0.99)	0.87 (0.68–1.10)	1.91 (0.79–4.60)
Bipolar spectrum disorders	1.13 (0.69–1.87)	1.06 (0.77–1.46)	1.12 (0.90–1.39)	2.13 (1.07–4.24)
Depressive disorders	1.16 (0.94–1.44)	1.05 (0.90–1.23)	1.03 (0.91–1.16)	1.94 (1.36–2.78)*
Anxiety disorders	1.09 (0.72–1.66)	1.24 (0.97–1.57)	0.99 (0.80–1.22)	1.76 (0.91–3.41)
PTSD and other stress related disorders	0.81 (0.43–1.53)	0.86 (0.64–1.16)	1.12 (0.81–1.54)	1.69 (0.54–5.27)
Eating disorders	0.60 (0.14–2.46)	5.95 (1.56–22.63)*	7.15 (0.72–70.72)	‐
Borderline personality disorder	1.16 (0.82–1.63)	1.33 (0.93–1.91)	1.22 (0.87–1.71)	‐
Other personality disorders	1.18 (0.82–1.71)	1.21 (0.90–1.65)	1.27 (0.98–1.64)	6.92 (1.01–47.21)
Any other psychiatric disorder	1.28 (0.99–1.66)	0.79 (0.58–1.07)	1.06 (0.77–1.45)	0.95 (0.26–3.48)

^a^ Alcohol use disorders: F10, substance use disorders: F11‐19, schizophrenia spectrum disorders: F20‐29, bipolar spectrum disorders: F30‐31, depressive disorders: F32‐39, anxiety disorders: F40‐42, PTSD and other stress related disorders: F43, eating disorders; F50, borderline personality disorder: F60.3, any other personality disorder: F60‐69 excluding F60.3, any other psychiatric disorder: F44‐48, F51‐59, F70‐99.

^b^ Controlled for previous psychiatric disorder, length of hospital stay, previous DSH episodes, method of DSH, marital status, education, income and ethnicity.

## 4 DISCUSSION

### 4.1 Main findings

This national study is the first, to our awareness, to provide an overview of general and clinical characteristics of DSH repeaters by gender and age group as well as giving a detailed account of the characteristics across the repeated episodes. Borderline personality disorder, depressive disorders, alcohol use disorder and other substance use disorders increased the risk of DSH repetition, but with large differences between age and gender subgroups. Additionally, prior history of hospital treated DSH significantly increased the risk of future DSH repetition. Differences were found between non‐repeaters of DSH and repeaters at their first episode with repeaters having higher psychiatric morbidity and lower socioeconomic status.

### 4.2 Characteristics of DSH repeaters and high‐volume repeat episodes

We found that 21.8% of the cohort presented to hospital with one or more repetitions, which corresponds well with the frequency of repetition estimated in a meta‐analysis of hospital treated DSH.^3^ Although DSH is known to be more common among females,^5^ this study is the first to demonstrate an increasing proportion of females with increasing number of repetitions; almost 90% of the group with 10 repetitions or more were females. While depressive disorders declined in prevalence with increasing numbers of repetitions, borderline personality disorder increasing from 2.9% among the repeaters at index episode of DSH to 42.2% among the high‐volume repeat episodes.

### 4.3 Borderline personality disorder

Borderline personality disorder yielded the strongest overall association with DSH repetition in the present study, and the increased risk was confined to females under the age of 40. These findings are in line with the established evidence of the strong link between borderline personality disorder and DSH repetition,^12^ but our study demonstrates well how much an increasing volume of DSH repetition is linked to this psychiatric disorder. DSH patients with borderline personality disorder need a thorough psychiatric evaluation and initiation of adequate evidence‐based treatment and follow‐up^13^ to break the pattern of DSH repetition. It has previously been shown that the quality of after care for DSH patients in Norwegian general hospitals varies greatly between institutions,^14^ which is a serious challenge to providing effective treatment for people with a pattern of DSH. Evidence‐based treatment proven to reduce the risk of DSH repetition exists.^15^ However, clinical guidelines ensuring that DSH patients, and DSH repeaters in particular, are offered this treatment are strongly needed.^16^

### 4.4 Alcohol use disorder and substance use disorders

Alcohol use disorder increased the risk of DSH repetition most prominently in males aged 65 and above. Individuals in need of treatment for alcohol use disorder have previously been linked to DSH repetition and suicide completion as well as death by any other cause.^17^ Consequently, more systematic follow‐up treatment of male DSH patients with alcohol use disorder shortly after discharge has a large potential of preventing both DSH repetition and death. However, noncompliance with after‐care is a problem in in this patient group,^18^ thus clinical approaches to strengthen patients' adherence to and engagement in treatments should be implemented. The increased risk of DSH repetition associated with substance use disorders in the present analysis was confined to males aged 25–39 and 65 and above. This corresponds well with prior research linking substance use disorders to hospital treated DSH repetition among adults^19^ and young adult males in particular.^20^

### 4.5 Affective disorders

Depressive disorders increased the risk of repetition among the elderly in the present cohort, while bipolar spectrum disorders increased the risk of DSH repetition in elderly females. As previous studies have shown that both bipolar and unipolar depressive disorders are associated with increased risk of suicide following DSH,^21^ psychiatric evaluation and follow‐up of this patient group is essential.

The lack of an overall association between depressive disorders and DSH repetition in the present cohort corresponds with previous findings from analysis of hospital data.^19^ The confinement of depressive disorders to single or few repetitions may reflect that this patient group is offered adequate follow‐up and that the offered treatment is both accepted by the patient and effective in reducing DSH repetition. Alternatively, the finding may be linked to the fact that borderline personality disorder often co‐occur with depression,^22^ but that since depression in borderline personality disorder often has a different clinical manifestation than depression in the absence of borderline personality disorder, depression may be more difficult to diagnose.^23^ We have no data to make a study of this question possible, but we cannot rule out the possibility that the true prevalence of depressive disorders in our cohort might be higher than the numbers show.

### 4.6 Other clinical factors important to DSH repetition

Previous history of DSH increased the risk of future DSH repetition. Higher number of previous DSH episodes yielded stronger associations with future repetition in both females and males. The seven‐fold risk of DSH repetition associated with ten repetitions or more in the present study is comparable to the report of a six‐fold risk of repetition associated with four previous episodes.^5^

### 4.7 Strengths and limitations

The index DSH episode in the data may not be the true first episode for the individual. As in previous register based studies on DSH, data on suicide intent are not available, thus we were unable to distinguish suicidal behavior from non‐suicidal self‐injury. Psychiatric disorders may be underdiagnosed in general hospitals. Consequently, some associations between psychiatric morbidity and risk of DSH repetition may not have been detected and some of our findings may be underestimates of the true risk.

This study is, to our awareness, the largest register study to date examining the association between psychiatric disorders and DSH repetition with data covering an entire national adult population of DSH patients treated in general hospital. By investigating psychiatric disorders through ICD‐10 codes, precise and objective information is provided.

## 5 CONCLUSION AND IMPLICATIONS

The present analysis shows that DSH repeaters are a complex and heterogeneous group. DSH repeaters were more often female, had lower socioeconomic status and higher psychiatric morbidity compared with non‐repeaters. History of previous DSH episodes displayed a strong association with future DSH repetition. Females with borderline personality disorder had the highest volume of DSH repetitions in the present cohort, and should therefore be referred to specialized treatment, especially if they have a history of prior DSH. This patient group is in need of a well‐designed treatment protocol according to updated clinical guidelines. In males over the age of 40, alcohol use disorder increases the risk of repetition and substance use disorders increased the risk among males aged 25–39 and over 65. Immediate follow‐up of this patient group after discharge is essential in prevention of future DSH repetition and the follow‐up intervention should focus on treatment engagement and prevention of early drop‐out. Depressive disorders were common among those with none or few repetitions. This could be explained by successful treatment, or could be linked to underdiagnosing of depressive disorders in the face of personality disorders. However, affective disorders increased the risk of DSH repetition among the elderly. In addition to the variables investigated in this paper, the risk of DSH repetition may also be affected by suicide intent. As we have investigated DSH, a term covering both suicidal behavior and non‐suicidal self‐harm, the risk factors for repetition identified by the present analyses also apply to DSH in the presence or absence of suicide intent. DSH repeaters form an easily identifiable patient group to clinicians in somatic hospitals that should be referred to psychiatric treatment. There is a strong need for clinical guidelines ensuring that DSH patients, and DSH repeaters especially, are offered specialized treatment and follow‐up. Intensive and specialized treatment should be given patients at the highest risk of repetition. In the face of limited health care resources, the in‐depth knowledge of age and gender specific risk factors provided by the present analysis will aid clinicians in tailoring treatment to specific groups.

## CONFLICT OF INTEREST

The authors have no conflicts of interest to decleare.

### PEER REVIEW

The peer review history for this article is available at https://publons.com/publon/10.1111/acps.13503.

## Data Availability

This study was based on individual‐level data from The Norwegian Cause‐of‐Death Register (held by Norwegian Institute of Public Health), The Norwegian Patient Registry (held by Norwegian Directorate of Health), The Central Population Register and the Statistics Norway's events database (held by Statistics Norway). The ethical approval of this research project does not include permission to publicly share the raw data. Qualifying researchers can apply for access to relevant data with the Norwegian Institute of Public Health (https://www.fhi.no/en/), the Statistics Norway (https://www.ssb.no/en/omssb/tjenester-og-verktoy/data-til-forskning) and Norwegian Directorate of Health (https://helsedirektoratet.no/english/norwegian-patient-registry) upon the approval from the Regional Committees for Medical and Health Research Ethics (https://helseforskning.etikkom.no/).
